# A Novel Source of Chest Pain in the Emergency Department

**DOI:** 10.5811/cpcem.2016.12.32030

**Published:** 2017-03-14

**Authors:** Ibrahim Isa, Jeffrey S. Bush, Simon Watson, Joseph Schoepf

**Affiliations:** * Medical University of South Carolina, Department of Emergency Medicine, Charleston, South Carolina; † Medical University of South Carolina, Department of Radiology, Charleston, South Carolina

## Abstract

This is a case report of a patient with an unusual presentation of an inferior vena cava (IVC) filter migration with a delayed presentation, and without electrical or valvular abnormalities. We discuss considerations and potential complications from IVC filter placement from the emergency physician perspective.

## INTRODUCTION

This is a case report of a patient with an unusual presentation of an inferior vena cava (IVC) filter migration with a delayed presentation, and without electrical or valvular abnormalities. Familiarity with IVC filters and their potential complications are important considerations in the clinical evaluations of many emergency department (ED) patients.

## CASE REPORT

The patient is a 38-year-old male with a history of coronary artery disease, hypertension and morbid obesity status post gastric bypass who presented to the ED with a chief complaint of chest pain. He stated that the pain began insidiously approximately five hours before he presented to the ED. He characterized the pain as a “pricking” sensation in his right anterior chest that radiated to his right scapula. He rated the pain as a 3–4 severity on a 10-point pain scale. He stated that the pain was continuous and currently present. The pain was unrelated to exertion. He denied any associated symptoms including fever, chills, cough, dyspnea, dizziness, diaphoresis, nausea, vomiting and diarrhea.

The patient had a past medical history significant for obesity, hypertension, hyperlipidemia, gastroesophageal reflux disease, deep venous thrombosis (DVT) and two prior ischemic strokes. Previous work-up of his prior cerebrovascular accidents revealed a patent foramen ovale, believed to be the underlying etiology. He denied congestive heart failure and prior myocardial infarction. Surgical history included gastric bypass three years earlier at which point an IVC filter had been placed. The IVC filter was never removed. However, after the procedure, a trans-esophageal and trans-thoracic echocardiogram, chest radiograph (CXR), magnetic resonance imaging/angiography of the abdomen and pelvis, and ultrasound of the aorta, IVC, and iliac vessels were all negative for the IVC filter*.* Per the medical record, it was concluded that the filter never deployed because it could not be located with the above imaging.

On initial presentation, the patient was conversational and in no acute distress. Physical exam revealed a well appearing, obese male who appeared his stated age with normal vital signs. A nine-system exam was unremarkable. The lungs were clear to auscultation bilaterally. The cardiac exam demonstrated regular rate and rhythm with no audible murmur, rub, or gallop. He was not diaphoretic, his jugular veins were flat and he had equal pulses without edema in his lower extremities. The chest, flank and back were not tender to palpation.

The patient declined pain medications. A chest pain work-up was done, which included a CXR, complete blood count, basic metabolic panel, troponin and an electrocardiogram (EKG).

Portable CXR showed stable cardiomegaly, interpreted by radiology to be consistent with prior radiographs. His EKG was normal without evidence of ST segment changes or T wave abnormalities. Bedside troponin was 0.00. Given that the patient had significant risk factors, we decided to perform a computed tomography triple rule-out (CT TRO) study of the chest to rule out pulmonary embolism (PE), aortic disease and to assess for coronary artery disease. The CT showed no evidence of significant coronary artery disease, pulmonary embolus, or acute aortic injury; however, it did demonstrate an IVC filter lodged in the right ventricle, seemingly adherent to the moderator band and right ventricular trabeculation. The patient’s CT was reconstructed to create the image below (Image) in which the IVC filter is clearly seen lodged in the right ventricle.

The patient was subsequently admitted into the cardiology service and multiple consultations were obtained. Interventional radiology and interventional cardiology did not believe they could safely extract the filter percutaneously due to concerns that they would injure the tricuspid valve. Cardiothoracic (CT) surgery recommended that the device be extracted via an open procedure that included sternotomy, cardiopulmonary bypass, and a brief period of cardioplegic arrest. The patient requested a second opinion from a CT surgery consultant who also recommended that the filter be removed. The patient had not yet made a decision and was subsequently discharged home on warfarin while considering his options.

## DISCUSSION

In 1973 Dr. Lazar Greenfield introduced his IVC filter permitting continuous flow with blockade of distal emboli. Since then, these devices have seen expanded use and development in an effort to prevent potentially fatal pulmonary emboli (PE). Their indications have expanded and now include patients undergoing bariatric surgery who are considered high risk for post-surgical DVT. In 1994 an article by BA Smith – “Vena Cava Filters” in *Emergency Medical Clinics of North America* – recognized that “the patient with a caval filter poses a challenge to the emergency physician.”^1^ In 1999 an estimated 49,000 IVC filters were placed.^2^ Since then, the use of the IVC filter has continued to expand and increase. The early 2000s saw the development of retrievable IVC filters, which extended the indication for placement to prevent vaso-thrombotic events in high-risk patients before surgery, including bariatric surgery. An estimated 220,000 bariatric surgeries were performed in 2009.^3^ Clearly, the number of IVC filters will continue to increase and so will the incidence of complication.

There is little emergency medicine literature discussing IVC filter-related complications; however, the surgery and vascular surgery literature over the last two decades outlines a number of complications. There are multiple cases of IVC filter migration to the right atrium or ventricle, as in the patient above.^4^,^5^,^6^ While there are similar reports in the literature, most of these have presented shortly after the IVC filter was placed. Also, most previous reports have focused on cardiac electrical irregularities caused by the IVC filter migration while this patient had a normal EKG. It is unclear why the initial imaging failed to reveal the IVC filter location. A possible explanation is that the filter had not yet migrated to the right ventricle at the time of the echocardiogram. CXR performed on the day the patient presented to the ED likely failed to reveal the presence of the filter as it was overlying the spine on the AP projection. Patients can have recurrent or “missed” PE in 2–4% of cases. There can also be delayed fracture of pieces of the filter, which can migrate and potentially cause perforations.^7^ There is also the long-term risk of direct erosion and perforation of the IVC itself.^8^ Additional complications can include IVC thrombus formation at the filter site and some other more immediate surgical issues including perforation, intimal tears, and air emboli.

In 2010 the FDA released a safety warning aimed at emergency medicine and surgery specialties. Since 2005 the FDA has received 921 device adverse-event reports involving IVC filters, of which 328 involved device migration, 146 embolizations (detachment of device components), 70 perforations of the IVC, and 56 filter fractures. Some of these events led to adverse clinical outcomes in patients. These types of events may be related to a retrievable filter remaining in the body for long periods of time, beyond the time when the risk of PE has subsided. The FDA is concerned that these retrievable IVC filters, intended for short-term placement, are not always removed once a patient’s risk for PE subsides.^9^

A limited, non-systematic literature search suggests the FDA is right; many retrievable filters are not actually removed, and these filters may then be associated with the aforementioned complications. Published work in peer-reviewed journals has reported technical success rates for removing filters ranging from 70–100%. However, reviews of actual clinical practice reveal retrieval rates as low as 22%.^10^ A systematic review in 2011, which included 37 studies and 6,834 patients, suggests that the mean retrieval rate was 34% and that “most of the filters became permanent devices.”^11^

## CONCLUSION

For emergency physicians there are a few significant pearls to remember about IVC filters. As IVC filter placement increases, the incidence of IVC filters and their complications will increase. Unfortunately, IVC filters do not totally preclude the incidence of PE. In fact, the PREPIC (Prevention du Risque d’Embolie Pulmonaire par Interruption Cave) study randomized 400 patients with DVTs to either an IVC filter or no-filter group and found no significant difference in fatal pulmonary emboli and overall survival at eight-year follow-up.^12^ There are multiple IVC filter-related complications that can cause significant abdominal and cardiopulmonary morbidity. If a patient has received an IVC filter, ask if it was ever removed. Many migrated IVC filters are found incidentally; be sure to arrange close follow-up in otherwise asymptomatic patients.

## Figures and Tables

**Image:**
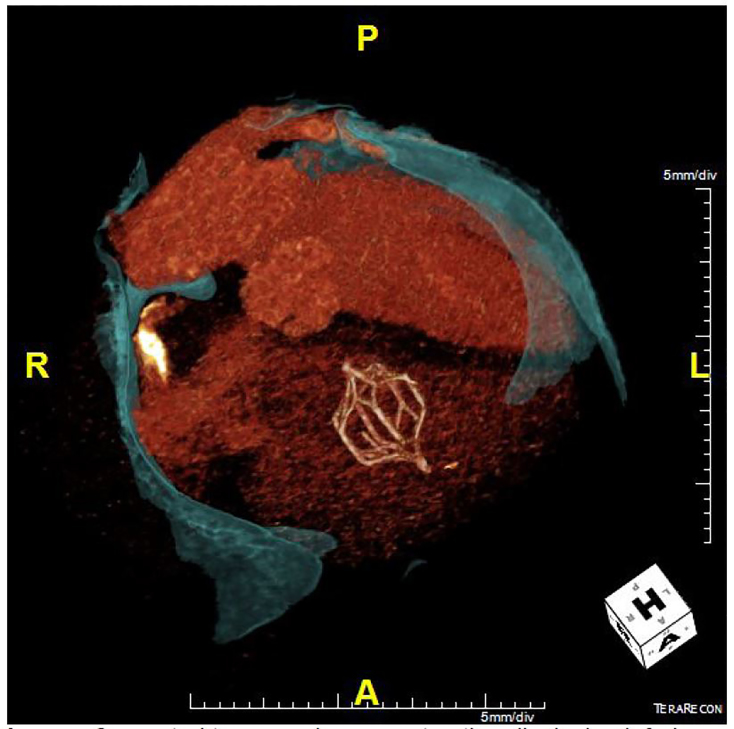
Computed tomography reconstruction displaying inferior vena cava filter lodged in right ventricle
